# A mixed‐methods study of staff perspectives on the barriers and facilitators to the implementation of patient‐reported routine outcome measures and feedback in alcohol and other drug treatment

**DOI:** 10.1111/dar.14007

**Published:** 2025-02-10

**Authors:** Nina Pocuca, Gabrielle Campbell, Anthony Barnett, Alison K. Beck, Rhiannon Ellem, Catherine A. Quinn, Peter J. Kelly, Briony Larance, Amanda L. Baker, Jason P. Connor, John Marsden, Gary C. K. Chan, Luke Connelly, Sabrina Lenzen, Michael Farrell, Robert Stirling, Suzie Hudson, Leanne Hides

**Affiliations:** ^1^ National Centre for Youth Substance Use Research, School of Psychology, Faculty of Health and Behavioural Sciences The University of Queensland Brisbane Australia; ^2^ National Drug and Alcohol Research Centre, UNSW Sydney Sydney Australia; ^3^ School of Psychology, University of Wollongong Wollongong Australia; ^4^ Discipline of Psychiatry, School of Medicine, The University of Queensland Brisbane Australia; ^5^ Addictions Department School of Academic Psychiatry, Institute of Psychiatry, Psychology and Neuroscience, King's College London London UK; ^6^ Department of Health and Social Care, Addiction and Inclusion Office for Health Improvement and Disparities London UK; ^7^ Centre for the Business and Economics of Health, The University of Queensland Brisbane Australia; ^8^ Department of Sociology and Business Law The University of Bologna Bologna Italy; ^9^ Network of Alcohol and Other Drugs Agencies Sydney Australia; ^10^ Drug Policy Modelling Program Social Policy Research Centre, UNSW Sydney Sydney Australia; ^11^ Centre for Alcohol and Other Drugs, NSW Ministry of Health Sydney Australia; ^12^ Lives Lived Well Brisbane Australia

**Keywords:** drug and alcohol treatment, implementation, patient‐reported outcome measures (PROM) and feedback, staff perspectives

## Abstract

**Introduction:**

Preliminary evidence supports the use of patient‐reported outcome measures (PROM) and feedback for enhancing client outcomes in alcohol and other drug (AOD) treatment. However, successful implementation remains challenging. This mixed‐methods study applied the Consolidated Framework for Implementation Research (CFIR) framework to examine inner setting and staff characteristics that act as barriers and facilitators to the implementation of PROMs in AOD treatment.

**Methods:**

To understand CFIR‐informed barriers and facilitators to implement PROMs in AOD treatment, qualitative interviews were conducted with *N* = 23 AOD counsellors. A separate quantitative survey was conducted with *N* = 108 AOD counsellors.

**Results:**

Four qualitative themes emerged: (i) PROMs and feedback are valuable to AOD treatment; (ii) counsellor resistance towards PROMs and feedback is a barrier to successful implementation; (iii) competing interests and logistical issues are barriers to the implementation of PROMs and feedback; and (iv) PROMs are a burden to clients that may serve to disengage them from treatment. Survey results indicated a positive association between leadership support (CFIR inner setting construct) and counsellor knowledge and beliefs regarding PROMs and feedback (CFIR staff characteristics construct; *β* = 0.35, 95% CI [0.13, 0.60]). Findings demonstrated a positive association between available PROMs resources (CFIR inner setting construct) and both knowledge and beliefs regarding PROMs and feedback (*β* = 0.31, 95% CI [0.14, 0.48]) and self‐efficacy to implement PROMs and feedback (CFIR staff characteristics construct; *β* = 0.18, 95% CI [0.04, 0.32]).

**Discussion and Conclusions:**

Findings point to the critical need to adopt a whole‐of‐organisation approach to foster buy‐in for PROMs and feedback to support successful implementation.

**Key Points**

- This study applied an implementation science framework to understand the barriers and facilitators to the implementation of patient‐reported outcome measures (PROM) and feedback in alcohol and other drug (AOD) treatment, from the perspective of AOD staff.
- Most AOD staff viewed PROMs and feedback as beneficial to AOD treatment, but identified several barriers to implementation.
- Implementation barriers included perceived burden of PROMs and feedback to AOD clients (Consolidated Framework for Implementation Research staff characteristics barriers) and competing interests and logistical issues (Consolidated Framework for Implementation Research inner setting barriers).
- A whole‐of‐organisation approach, including leadership and provision of resources, is needed to support successful implementation.

## 1 INTRODUCTION

A large body of evidence has positioned patient‐reported outcome measures (PROM) (i.e., the regular measurement of select variables to assess and monitor client treatment progress) and feedback to clinicians and/or clients, as a cost‐effective method for enhancing client outcomes in mental health treatment [1, 2]. Preliminary evidence also supports the use of PROMs and feedback in alcohol and other drug (AOD) treatment, with a systematic review finding reductions in AOD use (particularly among clients not progressing through treatment as anticipated), and improvements in client functioning, program participation and treatment success [3]. Despite emerging support for PROMs and feedback in AOD treatment, successful implementation remains a major challenge [4], highlighting the need for research identifying the barriers and facilitators to implementation.

Given the barriers to implementation may arise at the organisation, service‐provider and client‐level [5], research examining barriers and facilitators to implementation needs to consider the perspective of these different stakeholders. However, a recent scoping review by Migchels et al. [4] found only five studies have specifically examined staff perspectives on barriers and facilitators to PROMs and feedback in AOD treatment settings. These studies identified leadership support, an integrated patient record and regular PROM feedback to AOD staff as the greatest facilitators to implementation, while treatment dropout and burden to staff and patients were the greatest barriers to implementation [4]. While these studies provide important insights into the barriers and facilitators to PROMs and feedback implementation in AOD treatment, none of these studies applied an implementation science framework to interpret or scaffold results and guide future successful implementation [6].

The *Consolidated Framework for Implementation Research* (CFIR) is an implementation science framework often used to guide the successful implementation of healthcare interventions into practice [6, 7]. The CFIR includes 5 major domains that cover 37 different underlying constructs which can act as barriers and facilitators to successful implementation of an intervention (see the work of Damschroder et al. [7] and Louie et al. [8] for summaries of CFIR constructs and domains). The five CFIR domains include:

1. *intervention characteristics*—aspects of an intervention that may impact implementation success (e.g., evidence quality; design quality);
2. *outer setting*—external influences on intervention implementation (e.g., the economic, social and political context within which an organisation resides; external policies and incentives);
3. *inner setting*—characteristics of the implementing organisation influencing implementation (e.g., the structural and cultural context through which the implementation process proceeds; leadership engagement);
4. *characteristics of individuals*—(given AOD staff are the focus of the present paper, this domain is herein referred to as *staff characteristics*, for clarity); and
5. *process of implementation*—stages of implementation (e.g., planning; reflecting and evaluating).

The barriers and facilitators to implementing PROMs and feedback in AOD treatment identified in the Migchels et al. [4] review can be categorised under the *leadership engagement* and *available resources* constructs which form part of the *inner setting domain* of the CFIR framework. However, beyond the *inner setting domain*, a systematic review applying the CFIR framework to understand the implementation of evidence‐based practice in AOD treatment also identified the importance of *staff characteristics* on implementation success [8]. That is, of the five identified studies that examined the impact of staff characteristics on the implementation of evidence‐based practice in AOD treatment, four found *staff beliefs and attitudes* towards evidence‐based practice and staff *self‐efficacy* or confidence in their ability to deliver evidence‐based practice—constructs related to the CFIR *staff characteristics domain*—significantly enhanced implementation outcomes [8]. Therefore, given the impact of staff characteristics on the successful implementation of evidence‐based practice in AOD treatment, further research is needed to understand how staff characteristics may influence the implementation of PROMs and feedback in AOD treatment.

Beyond understanding how different CFIR inner setting and staff characteristics constructs may influence the implementation of PROMs and feedback in isolation, research is also needed to examine how these CFIR constructs may influence each other. For instance, research in other areas found greater leadership support for evidence‐based practice is associated with greater staff knowledge and more positive beliefs surrounding evidence‐based practice [9], while greater access to teaching‐related resources is associated with greater self‐efficacy for teaching [10]. Ultimately, these studies point to associations between CFIR constructs of *leadership support* (CFIR *inner setting construct*) and *staff knowledge and beliefs* (CFIR *staff characteristics constructs*), and *resource availability* (CFIR *inner setting construct*) and *self‐efficacy* (CFIR *staff characteristics constructs*). Given these findings, further research is needed to examine the association between these CFIR constructs within the context of implementing PROMs and feedback in AOD treatment. Ultimately, an increased understanding of the relationship between these constructs may facilitate the development of effective implementation strategies for the integration of PROMs and feedback in AOD treatment.

This mixed‐method study aimed to apply the CFIR *inner setting* and *staff characteristics domains* to: (i) understand staff perceptions of the barriers and facilitators to implementing PROMs and feedback in AOD treatment; and (ii) examine the association between *leadership support* and *available resources* for PROMs (constructs relating to the CFIR *inner setting domain*), and *knowledge and beliefs* regarding PROMs and *self‐efficacy* to implement PROMs and feedback (constructs relating to the CFIR *staff characteristics domain*). Relevant to the second aim, and based on research conducted in other areas [9, 10], it was hypothesised that: (i) greater leadership support for PROMs would be associated with more positive staff knowledge and beliefs regarding PROMs and feedback; and (ii) greater PROMs resource availability would be associated with greater self‐efficacy to implement PROMs and feedback.

## 2 METHOD

### 2.1 Design and setting

A mixed‐methods design (a qualitative phase followed by a quantitative phase) was used to explore staff perspectives on the barriers and facilitators to implementing PROMs and feedback into AOD treatment. This project was approved by the *Human Ethics Research Committee* at The University of Queensland (REF: 2022/HE000827; 2022/HE002235).

All data were collected from *Lives Lived Well* (LLW), a large not‐for‐profit provider of outpatient and inpatient AOD treatment (predominantly counselling) services in Australia. Since April 2020, LLW has collected PROMs electronically, at enrolment, 1 and 3 months. The PROMs that are collected from clients (refer to Table S1 for PROMs instruments and schedule) are completed via Qualtrics. The PROMs measures are part of the *International Consortium for Health Outcome Measurement's* recommended set of measures for capturing meaningful outcome domains for clients accessing substance use treatment services [11] and take approximately 20 min to complete. Clients complete PROMs either by themselves or with their AOD counsellor during treatment if not independently completed before attending treatment. Clients were automatically sent an invitation via SMS and email (along with two reminders, if required), to complete a follow‐up survey at 1 and 3 months, regardless of whether they were still enrolled in treatment. Once each PROM survey was completed, client survey data was automatically scored and feedback was sent to the treating counsellor (for an example of Qualtrics feedback delivered to staff, refer to Figure S1).

As part of the qualitative phase, interviews were conducted between August and September 2022. This was followed by a quantitative phase using a survey to collect data between January and October 2023. Data collection occurred prior to the organisation‐wide rollout of a brief personality‐targeted intervention. The intervention included motivational interviewing and coping skills training targeting a client's predominant personality risk profile, which also included the provision of PROM feedback.

### 2.2 AOD staff interviews

#### 2.2.1 Participants and procedure

A convenience sample of *N* = 133 client‐facing LLW AOD staff was invited (via email by a service manager) to participate in interviews aimed at better understanding the barriers and facilitators to PROMs and feedback in AOD services. In total, *N* = 23 LLW AOD counsellors (18 female; mean age = 42.83 years, SD = 11.45) across 11 LLW AOD services including both urban and rural/regional areas consented to participate in the study and completed an interview. Semi‐structured interviews were conducted by members of the study team (NP, GC, RD) using an interview guide which included questions focused on understanding barriers and facilitators to the implementation of PROMs and feedback, based on CFIR inner setting and staff characteristics constructs. For instance, questions including ‘What do you think of the idea of asking people accessing AOD services to complete PROMs about their substance use?’, ‘In your opinion, what is the purpose/role of PROMs?’, and ‘Do you find PROMs feedback helpful for informing treatment planning?’ assessed staff knowledge and beliefs about PROMs and feedback (CFIR staff characteristics constructs). Questions designed to understand inner setting barriers/facilitators to implementation included ‘Have any circumstances at work impacted your motivation/willingness to follow up on your clients' completion of PROMs/provide PROMs feedback to clients?’ and ‘In your opinion, what circumstances (e.g., workload, your understanding of the feedback sheet) make it easier/more difficult for you to deliver PROMs feedback?’

Consent to audio‐record interviews (conducted via Zoom) was obtained from participants to allow for professional verbatim transcription. Interviews focused on participants' experiences with and knowledge and beliefs regarding PROMs and feedback in AOD treatment. Once transcribed, all interviews were checked for consistency with recordings, and de‐identified to preserve participant anonymity. Interview length ranged from 29 to 74 min (mean = 50.52, SD = 10.32) and they were conducted until data saturation was approached (where no new themes were emerging), determined via discussion between the interviewers and research team [12].

#### 2.2.2 Data analyses

Reporting is in line with the *Consolidated Criteria for Reporting Qualitative Research* (COREQ; see Table S2 for the COREQ checklist) [13]. Qualitative analyses were conducted using iterative categorisation [14, 15] and adopted the O'Connor and Joffe [16] method to evaluate intercoder reliability. NP first coded all interviews using a deductive approach (with the semi‐structured interview and CFIR framework both serving as a guide) and compiled a codebook. The second coder (MB) then used the codebook to independently code a random sample of 10 interviews. Cohen's kappa analyses were conducted to examine intercoder reliability across the 10 double‐coded interviews, with the coding framework refined when discrepancies between coders (i.e., kappa <0.41) were encountered [17]. All coding was conducted in NVivo (version 14). Once completed, three members of the study team (NP, CT, RE) discussed and derived preliminary themes from the data, which were refined by NP to derive the final themes.

##### The researchers

Three female members of the research team (authors NP, GC, and RD, see acknowledgments) trained in qualitative interviewing conducted all interviews. NP and GC had a PhD and worked as university‐based researchers, while RD had an honours degree in psychology and was involved in the project as a Master of Clinical Psychology student. While NP, GC, RE, CAQ, and LH worked within a team with established research collaborations with LLW, the interviewers had no pre‐existing relationships with participants. The interview schedule, data analyses, and data interpretation were influenced by the authors' background, including university‐level education (mostly PhDs), experience in AOD research (all authors) and AOD treatment (AKB, PK, ALB and LH), gained within a Western cultural context.

### 2.3 AOD staff survey

#### 2.3.1 Participants and procedure

*N* = 141 LLW AOD counsellors who worked in a client‐facing role, and were part of the PROMs rollout within their service, were invited to participate in a survey which sought to understand their knowledge and beliefs, self‐efficacy, perceived leadership support, and available resources for implementing PROMs and feedback. The present study comprises *N* = 108 (77%) LLW AOD counsellors (85 female; *M*_age_ = 40.61, SD = 11.37) who consented to participate in the study and completed the baseline survey.

#### 2.3.2 Measures

Given there are no existing established measures, survey questions (see Table S3) were designed by the research team to explore participants' views about PROMs relating to the following categories:

##### Knowledge and beliefs regarding PROMs and feedback

Ten questions required participants to rate on a scale from 1 (strongly disagree) to 7 (strongly agree), how much they agreed with statements about PROMs and feedback (e.g., ‘Outcome Measures take into consideration the needs and preferences of clients’). Responses were averaged to obtain a single score ranging from 1 to 7. This measure had good internal consistency (Cronbach's *α* = 0.87).

##### PROMs and feedback self‐efficacy

Seven items assessed how confident participants felt (1 [not confident] to 5 [very confident]) with aspects of PROMs and feedback (e.g., ‘motivating clients to complete outcome measures’; ‘providing feedback to a client using the Outcome Measures Feedback?’). Responses were averaged to obtain a single score ranging from 1 to 5. The PROMs and feedback self‐efficacy scale had good internal consistency (Cronbach's *α* = 0.87).

##### Leadership support for PROMs

Two items assessed organisational leadership and direct supervisor support for PROMs. These items were rated on a scale from 1 (strongly disagree) to 7 (strongly agree) and averaged to derive a single score denoting leadership support for PROMs, which had acceptable internal consistency (Cronbach's *α* = 0.76).

##### Available resources for PROMs

Participants rated on a scale from 1 (strongly disagree) to 7 (strongly agree) their level of agreement with the availability of six resources to deliver PROMs and brief interventions (e.g., equipment and materials) at their AOD service. Items were averaged to derive a single score denoting available resources to deliver PROMs and feedback, which had good internal consistency (Cronbach's *α* = 0.88).

##### Correlates

Correlates included in quantitative analyses were: age; sex (0 [male], 1 [female]); highest degree (1 [certificate], 2 [diploma], 3 [bachelors degree], 4 [master's degree]); and separate questions examining length of time working at LLW and length of time working in the AOD sector (0 [started within the past month], 1 [<1 year], 2 [1–2 years], 3 [2–3 years], 4 [3–6 years], 5 [≥6 years]).

#### 2.3.3 Data analyses

Three participants had missing data on leadership support and available PROMs resources. As there was no other missing data, missing data were deleted listwise. Multiple linear regressions examined the association between leadership support and available resources for PROMs: (i) knowledge and beliefs regarding PROMs and feedback; and (ii) self‐efficacy for implementing PROMs and feedback. Independent variables were standardised prior to analyses which were conducted in SPSS (version 29), with bootstrapping applied to account for any non‐normality.

## 3 RESULTS

### 3.1 AOD staff interviews

#### 3.1.1 Participant characteristics

Most participants had a background in counselling (30%) or social work (26%) and had worked an average of 5.7 years in the AOD treatment sector.

#### 3.1.2 Qualitative themes

Four themes were identified in the data: (i) PROMs and feedback are valuable to AOD treatment; (ii) counsellor resistance towards PROMs and feedback is a barrier to successful implementation; (iii) competing interests and logistical issues are barriers to the successful implementation of PROMs and feedback; and (iv) PROMs are a burden to clients that may serve to disengage them from AOD treatment. These themes and example quotes are provided below and in Table 1, where they are mapped onto their corresponding CFIR constructs and domains.

**TABLE 1:** Identified themes, associated codes and illustrative quotes.

Theme and CFIR construct and domain	Example quotes
**Theme 1: PROMs and feedback are valuable to AOD treatment** CFIR construct: Knowledge and beliefs about the intervention CFIR domain: Staff characteristics	‘[PROMs are] a bit of an opener to your discussion, so maybe the uncomfortable topics around suicidality … I use that to start the conversation so that it doesn't come out of nowhere. ‘I see for suicidality, you answered this. Can you tell me a bit more about what that looks like?’ so it's a great way to open up the discussion’. (Participant 5) ‘I love it as a tool of comparing even baseline and three months and being like, “When we first started, your PHQ‐9 score was 15, and now it's a three. Oh my God. What do you reckon has changed?” So it is a great starting point if you can get your clients to do it and can really add some value’. (Participant 3) ‘I'm using their scores as more of a conversation guide and a tool to maybe zone in on some areas that could be problematic for them that might help to provide them relevant referrals, whether that be to a GP or a domestic violence service …’ (Participant 6) ‘I guess [PROMs are] a self‐report from [clients] about a snapshot in time so I guess we sort of know what it's like when they commence service with us, and then we can use it when we check in with them at regular intervals to see if their mental health is being impacted in either a good or a non‐good way by treatment. It allows us to pick up clinical deterioration and that kind of thing’. (Participant 15)
**Theme 2: Counsellor resistance towards PROMs and feedback is a barrier to successful implementation** CFIR construct: Individual stage of change CFIR domain: Staff characteristics	‘[PROMs and feedback] makes it harder to move through treatment, especially when you have six sessions, and you get caught up with outcome measures for one, and then doing a bio in maybe a next session, and then them talking through everything. And it just kind of chews up the time when I think naturally you would kind of have moved on’. (Participant 6) ‘If [the client has completed PROMs], great. If they haven't, I just take that on board to go, “They're not in the space where they want to,” and besides, I'm asking questions about what their use looks like, what their mental health looks like, all that stuff anyway … I don't actually care whether it's done. I don't base my treatment around it’. (Participant 14) ‘All of its really valuable information, but that is information for us, I think the clients probably struggle to see the value [of PROMs] for them … It's really there for us to get funding, ultimately you know?’ (Participant 23)
**Theme 3: Competing interests and logistical issues are barriers to the successful implementation of PROMs and feedback** CFIR construct: Relative priority and available resources CFIR domain: Inner setting	‘Well, often, you have an idea of what you're going to do in the session, but [the client] might come to you in crisis, and the last thing they want to do is discuss outcome measure results. So it can be like—not even necessarily crisis, but that's not on their agenda. They want to talk about their feelings. They want to talk about what's happened. So I haven't really gotten into great practice of going through [outcome measure feedback] with them’. (Participant 13) ‘Well, I just think [outcome measure feedback] should be interactive … It shouldn't just be a wall of black and white text with some squares and stuff. I shouldn't have to look for the information’. (Participant 1) ‘If I want to compare—which I do—the previous measures to the current measures, there's no easy way. I have to have multiple screens open’ (Participant 17)
**Theme 4: PROMs are a burden to clients that may serve to disengage them from AOD treatment** CFIR construct: Knowledge and beliefs about the intervention CFIR domain: Staff characteristics	‘Some clients are more than happy to do [PROMs], other clients, couldn't really care less about those things. And those clients would be corrections clients who are forced, I suppose, coerced into coming to treatment’. (Participant 9) ‘[Clients] just don't want to do [PROMs]. I've got clients that have gone – every time I talk to them, “Oh, have you had a chance to do them measures?” Often, you'll get, “I've lost them,” or, “My phone doesn't—I can't open it,” and you resend. But yeah, there just seems to be a reluctance or hesitation to do them by the clients’. (Participant 16) ‘… they do complain sometimes about the length of how long it takes, so many questions’. (Participant 15) ‘… some [clients] live more on the farms and stuff. And I know where I am I'm kind of beachside and our reception here is terrible. So if they don't have Wi‐Fi at home and they're trying to access [outcome measure] links, a lot of them will struggle and just shut it and couldn't be bothered’. (Participant 12)

Abbreviations: AOD, alcohol and other durgs; CFIR, Consolidated Framework for Implementation Research; PROM, patient‐reported outcome measures.

##### Theme 1: PROMs and feedback are valuable to AOD treatment

This theme relates to staff knowledge and beliefs about the intervention (i.e., familiarity with and perceived value placed on the intervention), classified under the CFIR staff characteristics domain [7]. Most participants (*n* = 17) acknowledged the benefits of PROMs and feedback to AOD treatment, including: (i) providing AOD staff with a snapshot of a client's situation; (ii) facilitating dialogue between the counsellor and client; (iii) promoting client reflection and insight; and (iv) allowing AOD staff to track client progress across treatment. Namely, PROMs were seen as a valuable tool for getting a quick snapshot of a client's situation and discerning whether referral to other supports (e.g., domestic and family violence, gambling) was required.

‘[PROMs give] us a bit of a picture of how the client presents, where they're at, sort of cuts through a little bit of rapport work.' (Participant 20)

Additionally, the process of completing PROMs and feedback was seen as a useful way to initiate difficult conversations with clients about their AOD use, mental health, or suicidal ideation and bring client attention to relationships between these factors. When a client completed PROMs for the second or third time, participants noted that PROMs served as an opportunity to pick up on clinical deterioration or to acknowledge positive change.

‘, I think [PROMs are] a necessity in terms of tracking … progress or lack thereof so you can have a qualitative measure of progress. If there isn't progress, you can add additional supports …' (Participant 21)

##### Theme 2: Counsellor resistance towards PROMs and feedback is a barrier to successful implementation

This theme relates to the individual stage of change construct (i.e., the stage an individual is in as they progress towards skilled, enthusiastic and sustained implementation), classified under the CFIR staff characteristics domain [7]. Several participants developed a negative opinion of PROMs and feedback which presented a challenge for PROMs implementation. For instance, six participants noted that most of the information collected via PROMs was already collected through discussions with the client and perceived PROMs and feedback as too rigid for their style of practice. Thus, participants felt that the integration of PROMs and feedback provided little relative advantage to their therapeutic practice.

‘I've been actually really resistant [to PROMs]. I've got a problem with change, but also, I find it quite static …' (Participant 13)

Four participants also noted the complexity of PROMs and feedback as a barrier to implementation. That is, PROMs and feedback were viewed as another time‐consuming organisational requirement that contributed to an already heavy workload and diverted time away from delivering treatment to clients.

‘There's an expectation that we'll use a session to complete an outcome measure, which I think, while there's some really valuable data, it easily takes up the best part of a session, because most people will talk through it. When considering we only have five or six sessions, basically a session is gone …' (Participant 22)

Finally, 10 participants noted an incompatibility between aspects of PROMs and feedback with their own norms, values, and beliefs surrounding AOD treatment. That is, three participants viewed PROMs as primarily an organisational requirement to secure funding for their AOD service. Additionally, seven participants felt sending PROMs to clients who had left treatment was intrusive and may disengage clients from seeking AOD treatment in the future. Thus, these participants found PROMs to be at odds with their norms, values, and beliefs surrounding the need for client‐centred AOD treatment.

‘… I do have a bit of an ethical dilemma because we're required to do [PROMs] and so sometimes, I have to balance trying to fulfil that organisational requirement to get them done with what I know is going on for the client and their particular needs.' (Participant 17)

‘If I had someone who stopped engaging, I wouldn't feel comfortable calling them for outcome measures, specifically because I don't want to turn them off seeking help in the future. So if they feel like ‘I'm being bombarded’ or something like that, that's not what I want to do.' (Participant 6)

In summary, several participants developed the opinion that PROMs and feedback offered little relative advantage to their AOD practice, were time‐consuming and were incompatible with their own norms, values and beliefs surrounding AOD treatment, thus leading them to resist implementation.

##### Theme 3: Competing interests and logistical issues are barriers to the successful implementation of PROMs and feedback

This theme relates to the relative priority (i.e., perceived importance of and priority placed on implementation) and available resources (i.e., the time, training and education dedicated to implementation), CFIR constructs of the inner setting domain. Regardless of whether they adopted or resisted implementation (see Theme 2), many participants (*n* = 14) noted that a lack of time during treatment combined with competing interests and logistical issues precluded them from fully engaging with PROMs and feedback in their AOD practice. That is, participants highlighted the limited time they have with clients and that if a client came to treatment in a crisis, had a tough week, needed to prepare for an upcoming court appearance, or simply wanted to discuss something other than PROMs during their session, these competing interests were prioritised over PROMs and feedback.

‘… I've got to balance up, “I could [complete PROMs] in this appointment now to get a whole lot of information from the outcome measures that actually I already have most of it anyway”, or, “I could actually spend the time helping them with how they're going to manage their high‐risk situation that's coming up today”.' (Participant 17)

Participants also highlighted not having the appropriate resources to effectively deliver PROM feedback. That is, participants noted that they did not have an easy way of comparing baseline and follow‐up PROMs, which in turn meant that AOD staff had to spend more time organising PROM feedback into a more easily digestible format (e.g., with graphs), disengaging them and creating a barrier for the provision of PROM feedback.

‘I think it would be better if [PROM feedback] was presented in a different way … You know, you could print it off in a nicer way. Like I have my own little sort of spreadsheet thing that I put it all in.' (Participant 12)

##### Theme 4: PROMs are a burden to clients that may serve to disengage them from AOD treatment

As with theme 1, this theme relates to the knowledge and beliefs about the intervention construct, classified under the CFIR staff characteristics domain. Participants—regardless of their own personal view on PROMs and feedback—expressed a perceived lack of client motivation, ambivalence or resistance towards completing PROMs, and that insistence upon PROMs can lead these clients to disengage from AOD treatment. Particularly, participants expressed that clients who are court‐mandated to receive AOD treatment are more hesitant to complete PROMs, and cautious and concerned about who will see their PROMs results.

‘I have had some [clients] that just don't want to do [PROMs]. They just want to come in and talk to somebody … So I probably had three that have refused the service due to the outcome measures.' (Participant 12)

Additionally, the confronting nature of completing PROMs, particularly at service entry, was perceived as potentially disengaging for both court‐mandated and non‐mandated clients. Finally, most participants (*n* = 16) expressed that PROMs and feedback may be burdensome to clients given some questions are confusing or unclear (potentially due to client limited literacy), the PROMs are long and time‐consuming, and clients have limited access to the technology required to compete them (e.g., smart phone, the internet).

‘I've had some feedback of, “Oh, this looks like I'm a horrible person”, so I think [PROMs are] quite triggering.' (Participant 16)

‘I found a lot of clients can get confused by [the PROMs]. So if their reading/writing levels aren't very good and they can't understand what the question's asking.' (Participant 3)

### 3.2 AOD staff survey

#### 3.2.1 Participant characteristics

Most participants had worked within the AOD treatment sector for ≥3 years, had been working at LLW for ≥2 years and reported an average caseload of 16–20 active clients (ranging from >5 to 50+) at any one time. Other sample descriptive statistics are summarised in Table 2 (for all item‐level sample descriptive statistics related to staff views about PROMs please refer to Table S4, Supporting Information). Given the moderate correlation between variables examining length of time working at LLW and length of time working in the AOD sector (see Table S5, Supporting Information), only length of time working at LLW was retained in regression analyses.

**TABLE 2:** Staff characteristics and descriptive statistics from quantitative study (*N* = 108).

Variable	*M* (SD)/% (*n*)
Age, *M* (SD)	40.61 (11.37)
Female, % (*n*)	79% (85)
Highest degree^a^	
Certificate or diploma	29% (31)
Bachelors	53% (57)
Masters	18% (20)
Length of time working at AOD treatment service^a^	
<1 year	15% (16)
1–3 years	44% (48)
3+ years	41% (44)
Length of time working in the alcohol and other drug sector^a^	
1–3 years	29% (31)
3+ years	71% (77)
Knowledge and beliefs regarding PROMs and feedback	5.05 (0.93)
Self‐efficacy to implement PROMs and feedback	3.93 (0.67)
Leadership support for PROMs	6.37 (0.84)
Available resources for PROMs	5.26 (1.08)

Abbreviations: AOD, alcohol and other drugs; PROM, patient‐reported outcome measures.

^a^ Some categories were merged due to small cell size to preserve participant anonymity. Range for knowledge and beliefs regarding PROMs and feedback, Leadership Support for PROMs, and available resources for PROMs is 1 (strongly disagree) to 7 (strongly agree). Range for self‐efficacy to implement PROMs and feedback is 1 (not confident) to 5 (very confident).

#### 3.2.2 Regression analyses

Results for multiple linear regression analyses examining the association between leadership support and available resources for PROMs, and participant knowledge and beliefs regarding and self‐efficacy to implement PROMs and feedback, are reported in Table 3. Regression analyses revealed greater leadership support and availability of resources required for PROMs were associated with more positive participant knowledge and beliefs regarding PROMs and feedback. Additionally, greater availability of resources required for PROMs was also associated with greater self‐efficacy to implement PROMs and feedback.

**TABLE 3:** Multiple linear regression models examining the association between leadership support and available resources for PROMs, and participant knowledge and beliefs regarding and self‐efficacy to implement PROMs and feedback.

	Knowledge and beliefs regarding PROMs and feedback			Self‐efficacy to implement PROMs and feedback		
	*β* (SE)	[95% CI]	*p*	*β* (SE)	[95% CI]	*p*
Age	−0.12 (0.09)	[−0.33, 0.02]	0.168	−0.02 (0.07)	[−0.18, 0.1]	0.729
Sex	−0.09 (0.19)	[−0.48, 0.28]	0.626	−0.05 (0.16)	[−0.36, 0.26]	0.743
Highest degree	−0.05 (0.08)	[−0.21, 0.11]	0.566	−0.05 (0.06)	[−0.18, 0.06]	0.398
Length of time working at AOD service	−0.06 (0.08)	[−0.2, 0.12]	0.403	0.01 (0.07)	[−0.12, 0.15]	0.864
Leadership support for PROMs	**0.35 (0.12)**	**[0.13, 0.6]**	**0.002**	0.17 (0.1)	[0.00, 0.39]	0.083
Available resources for PROMs	**0.31 (0.08)**	**[0.14, 0.48]**	**0.001**	**0.18 (0.07)**	**[0.04, 0.32]**	**0.010**

*Note*: Significant effects are bolded.

Abbreviations: AOD, alcohol and other drugs; CI, confidence interval; PROM, patient‐reported outcome measures.

## 4 DISCUSSION

Informed by the CFIR implementation framework, this mixed‐methods study identified four themes in the qualitative data: (i) PROMs and feedback are valuable to AOD treatment; (ii) counsellor resistance towards PROMs and feedback is a barrier to successful implementation; (iii) competing interests and logistical issues are barriers to the successful implementation of PROMs and feedback; and (iv) PROMs can constitute a burden to some clients that may serve to disengage them from AOD treatment. Reflecting associations seen in other areas [9, 10, 18] and in line with hypothesised effects, the quantitative results underscore that greater leadership support is associated with greater knowledge and more positive beliefs regarding PROMs and feedback. In addition, available resources for PROMs are associated with greater knowledge and more positive beliefs and greater self‐efficacy to implement PROMs and feedback.

Most participants perceived PROMs and feedback as valuable to AOD treatment, a sentiment also reflected in other studies conducted with AOD treatment staff [19] and people receiving AOD treatment [20]. Despite this, several barriers to implementing PROMs and feedback categorised under the CFIR staff characteristics domain were identified. For instance, many participants developed a negative opinion of PROMs and feedback leading to resistance towards implementation. That is, several participants perceived PROMs and feedback as providing little advantage to their clinical practice, time‐consuming, and at odds with their own norms, values and beliefs regarding effective, client‐centred AOD treatment. These barriers map onto the innovation‐decision process model [21] which is championed in the CFIR framework for understanding the individual stage of change construct. Namely, the innovation‐decision process model [21] posits that staff opinions towards an intervention are shaped by the perceived relative advantage, complexity (i.e., time, scope, changes from usual practice), and compatibility of the innovation with staff members' own norms, values, and beliefs. In turn, staff opinions towards an intervention influence their decision to adopt or resist implementation of the innovation [7, 21]. Similar to the present study, Myers et al. [19] found initial resistance towards PROMs and feedback in their study of AOD treatment staff in South Africa, which softened once staff had an increased awareness of the benefits of PROMs and feedback to their clinical practice. This highlights the importance of training AOD counsellors in the application of PROMs and feedback to treatment to foster buy‐in.

Additionally, knowledge and beliefs regarding PROMs and feedback (CFIR staff characteristics construct) were identified as a barrier to implementation. Almost two‐thirds of participants perceived that clients were resistant to PROMs and that this could ultimately serve to disengage them from AOD treatment, a perception noted previously among AOD treatment providers [22]. This raises the issue about whether staff viewed PROMs as a reporting requirement that may not necessarily benefit client engagement in treatment. Among clients who are willing to complete PROMs, participants perceived that barriers including limited literacy would impede PROMs completion, an idea reflected by AOD treatment providers [22, 23] and some clients receiving AOD treatment [20, 24]. Despite these barriers, studies conducted with clients receiving AOD treatment (including those with limited literacy) show that they welcome completing PROMs, particularly when confidentiality is ensured [20, 24]. Further, a meta‐analysis of client experiences of PROMs in mental health treatment settings highlighted that many perceived PROMs as a way of actively collaborating on their treatment [25], underscoring the potential benefit of PROMs and feedback to collaborative AOD treatment. Ultimately, these findings highlight potential misconceptions among AOD counsellors regarding the client burden of PROMs and feedback that need to be addressed to facilitate successful implementation.

The qualitative findings demonstrated how logistical issues and a lack of available resources (CFIR inner setting construct) act as barriers to PROMs and feedback implementation. As highlighted in the quantitative findings, when supports and available resources for PROMs were available, staff attitudes tended to demonstrate greater knowledge and more positive beliefs concerning PROMs. Participants often discussed how lacking the time and user‐friendly PROMs feedback undermined the implementation of PROMs and feedback during treatment. Thus, given the limited time participants had with clients during treatment, many prioritised other competing interests over PROMs and feedback. Limited time has been identified as a barrier to implementing PROMs and feedback in other health treatment settings where PROMs were used [26]. In contrast, easy to understand PROMs feedback (e.g., colour‐coded and visualised using graphs) has been identified as an important facilitator of PROM feedback by both AOD staff and clients [23].

### 4.1 Practical implications

Adding to the facilitators to the integration of PROMs and feedback in AOD treatment identified by Migchels et al. [4], the present qualitative and quantitative findings highlight the need for a whole‐of‐organisation approach that encompasses support from leaders, effective training and the provision of adequate resources to facilitate the successful implementation of PROMs and feedback in AOD treatment. Effective training may focus on: (i) highlighting how PROMs and feedback fit with client‐centred care to enhance client treatment outcomes; (ii) highlighting clients' positive perceptions of PROMs and feedback to address misconceptions about client burden; and (iii) equipping counsellors with the skills required for PROMs and feedback. Beyond this, organisations should also strive to provide adequate resources for the implementation of PROMs and feedback. One way of achieving this may be to reduce the time‐burden of PROMs by providing alternate pathways for collection outside of treatment (e.g., online self‐report prior to treatment) and by designing an integrated PROMs feedback record that is user‐friendly for both AOD counsellors and clients. In turn, this may help to address the concern of PROMs and feedback constituting a reporting burden that may adversely impact client engagement in treatment.

### 4.2 Strengths and limitations

The present study has several strengths including the use of a mixed‐methods design and application of an established implementation framework (i.e., CFIR [7]), which allowed for a comprehensive examination of inner setting and staff characteristics that act as barriers and facilitators to implementing PROMs and feedback [4]. Validated survey instruments that explore staff views about PROMs may further benefit research in the future.

A limitation is that participants were recruited from a single, not‐for‐profit organisation that already had significant exposure to PROMs and feedback (given LLW have been implementing PROMs since 2020). Given the AOD treatment sector comprises of a mix of government, not‐for‐profit, and privately funded services staffed by providers from different backgrounds (e.g., counsellors, social workers, psychologists, peer workers, nurses, doctors) [27, 28, 29], how PROMs and feedback are viewed within different treatment contexts is not straightforward or easily predictable. Therefore, future research on the impact of outcome monitoring within local treatment settings, guided by critical social science [30], implementation science [6] and co‐design methods [31] with clients and clinicians, will be important.

Although this study provided insight into the barriers and facilitators to PROMs and feedback implementation in AOD treatment from the perspective of staff, future research should explore client perspectives, and how staff/client perspectives translate to objective implementation outcomes including the number of PROMs completed and feedback delivered to clients.

## 5 CONCLUSIONS

While many participants recognised the benefits of PROMs and feedback to AOD treatment, several staff characteristics (e.g., negative perceptions towards PROMs and feedback) and inner setting constructs (e.g., low relative priority and lack of time and available resources for PROMs and feedback) acted as barriers to implementation. Ultimately, findings highlight the need for a whole‐of‐organisation approach that fosters buy‐in for PROMs and feedback, to facilitate successful implementation.

## AUTHOR CONTRIBUTIONS

Each author certifies that their contribution to this work meets the standards of the International Committee of Medical Journal Editors.

## FUNDING INFORMATION

This study was funded by the National Health and Medical Research Council Meaningful Outcomes in Substance Use Treatment Centre of Research Excellence. NP, GC, AKB, and RE are funded by the National Health and Medical Research Council Centre of Research Excellence. LH was partially funded by Lives Lived Well. AB has previously received funding from Camurus AB for unrelated research. JM declares funding to King's College London from Indivior for the research costs of a multi‐centre randomised controlled trial of extended‐release buprenorphine versus the daily standard‐of‐care in England and Scotland (sponsor: King's College London and South London and Maudsley NHS Trust). MF has received an Indirect Educational Grant from Indivior, which is unrelated to the current research. None of the funding bodies were involved in the study design, collection, analysis, interpretation of results or writing of this publication.

## CONFLICT OF INTEREST STATEMENT

The authors declare no conflicts of interest.

## Supporting information


**Data S1.** Supporting Information.

## Data Availability

Research data are not shared.
