# Supplementary material for: A mixed‐methods study of staff perspectives on the barriers and facilitators to the implementation of patient‐reported routine outcome measures and feedback in alcohol and other drug treatment
Source: Drug Alcohol Rev. 2025 Feb 10;44(3):759–71. doi: 10.1111/dar.14007 (PMC11886491; doi:10.1111/dar.14007)
Supplement: Supplementary file 1 — Data S1. Supporting Information. [file DAR-44-759-s001.docx]

Table S1. Outcome measure schedule

| **Survey instruments** | **Assessment (Baseline)** | **1 month-follow up** | **3 months follow-up** |
| --- | --- | --- | --- |
| World Health Organization Alcohol, Smoking and Substance Involvement Screening Test (ASSIST) [1] | Yes |  | Yes |
| Australian Treatment Outcome Profile (ATOP) [2] | Yes | Yes | Yes |
| Patient Health Questionnaire (PHQ-9) [3] | Yes | Yes | Yes |
| Generalized Anxiety Disorder (GAD-7) [4] | Yes | Yes | Yes |
| Posttraumatic Stress Disorder (PTSD) screen [5] | Yes |  |  |
| Psychosis (“Has a doctor ever told you may have schizophrenia or a psychotic disorder?” No/Yes) | Yes |  |  |
| Problematic gambling was determined by using a cut-off score of 8 or more on the Problem Gambling Severity Index [6] | Yes |  | Yes |
| Patient Experience Survey (PEQ) [7] |  | Yes | Yes |

**References**

1. WHO ASSIST Working Group. The alcohol, smoking and substance involvement screening test (ASSIST): development, reliability and feasibility. Addiction. 2002;97:1183-1194.
2. Lintzeris N, Mammen K, Holmes J, Deacon R, Mills L, Black E. Australian Treatment Outcomes Profile (ATOP) Manual 1: Using the ATOP with Individual clients. 2020. Available from: <https://www.seslhd.health.nsw.gov.au/sites/default/files/groups/Drug_Alcohol/ATOP2020.pdf>
3. Kroenke K, Spitzer RL, Williams JB. The PHQ‐9: validity of a brief depression severity measure. J Gen Intern Med. 2001;16:606-613.
4. Spitzer RL, Kroenke K, Williams JB, Löwe B. A brief measure for assessing generalized anxiety disorder: the GAD-7. Arch Intern Med. 2006;166:1092-1097.
5. Prins A, Bovin M, Kimerling R, Kaloupek D, Marx B, Pless Kaiser A, Schnurr P. The primary care PTSD screen for DSM-5 (PC-PTSD-5). Washington, DC: National Center for PTSD. 2022.
6. Miller NV, Currie SR, Hodgins DC, Casey D. Validation of the problem gambling severity index using confirmatory factor analysis and rasch modelling. Int J Methods Psychiatr Res. 2013;22:245-255.
7. Pettersen KI, Veenstra M, Guldvog B, Kolstad A. The Patient Experiences Questionnaire: development, validity and reliability. Int J Qual Health Care. 2004;16:453-463.

Figure S1: Example of qualtrics feedback


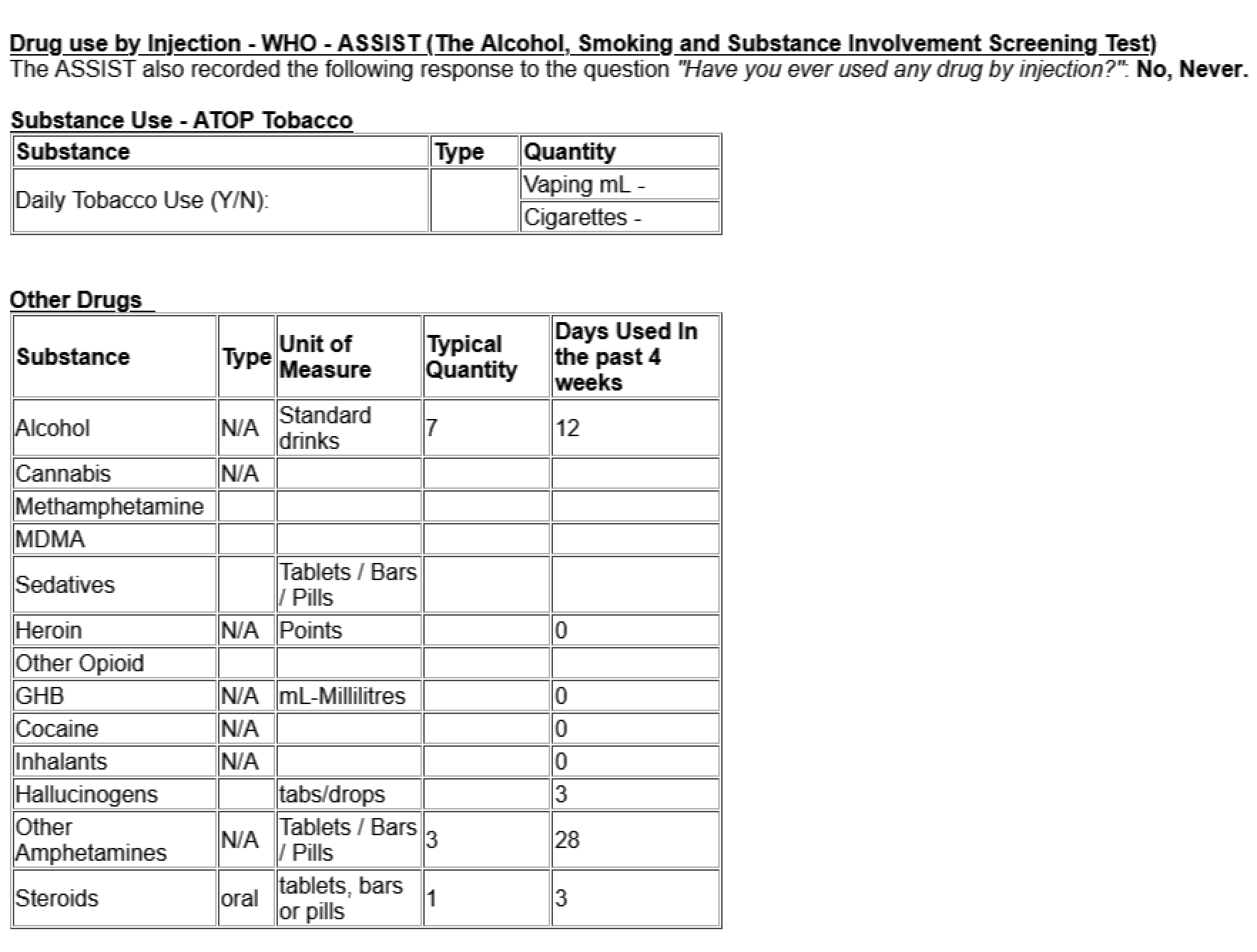

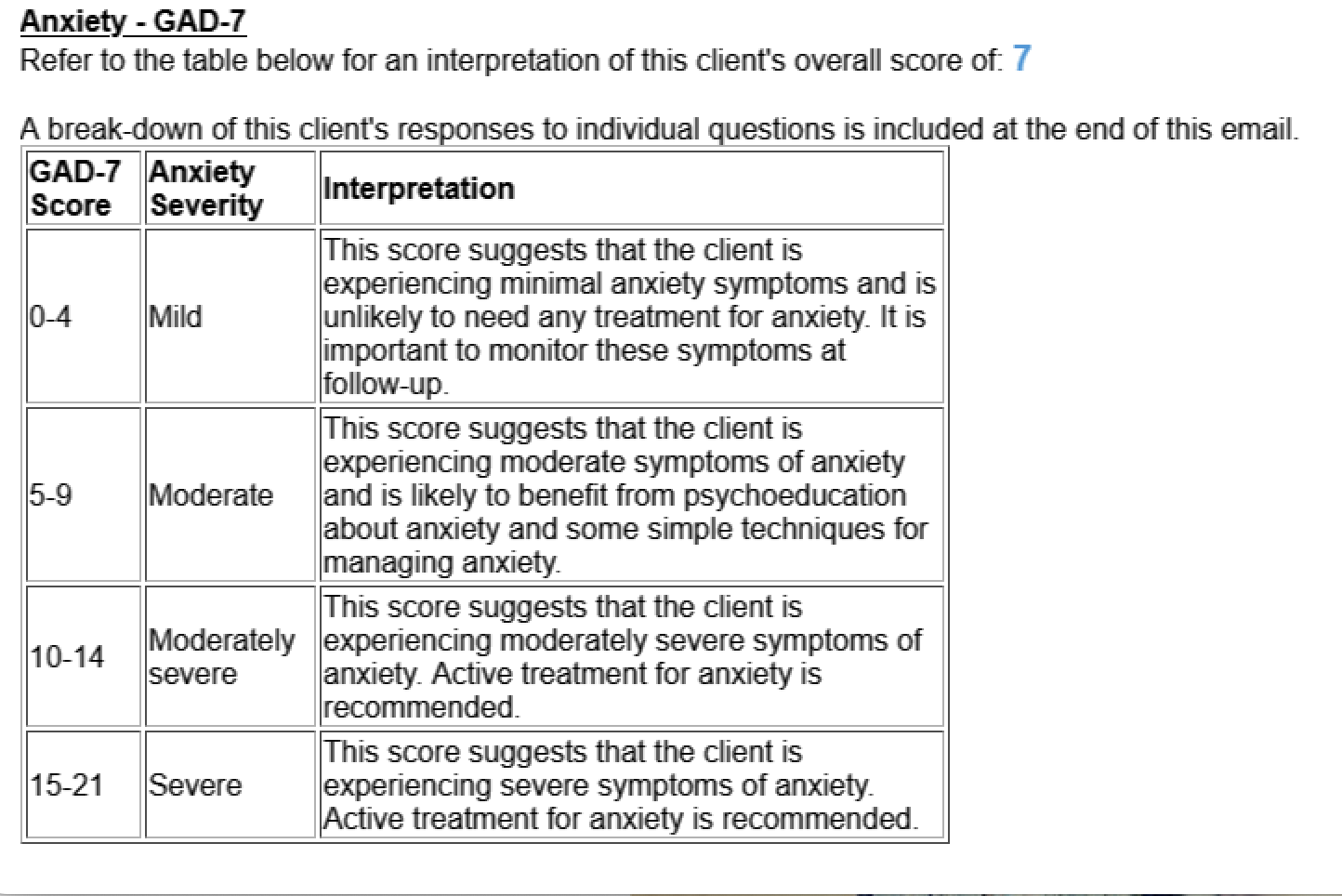


Table S2. Consolidated criteria for reporting qualitative studies (COREQ): 32-item checklist

| **Item number** | **Topic** | **Guide questions/description** | **Reported Page/s** |
| --- | --- | --- | --- |
| **Domain 1: Research team and reflexivity** | | |  |
| *Personal characteristics* | | |  |
| 1 | Interviewer/facilitator | Which author/s conducted the interview or focus group?  **NP, GC, and RD conducted the interviews.** | 10 |
| 2 | Credentials | What were the researcher’s credentials? E.g. PhD, MD  **NP – PhD**  **GC – Masters in Criminology, PhD**  **RD - BPsych (Hons)** | 11 |
| 3 | Occupation | What was their occupation at the time of the study?  **NP – Research Fellow**  **GC – Senior Research Fellow**  **RD – Master of Clinical Psychology student** | 11 |
| 4 | Gender | Was the researcher male or female?  **All interviewers were female.** | 11 |
| 5 | Experience and training | What experience or training did the researcher have?  **NP and GC had a PhD and worked as university-based researchers, while RD had an honours in psychology and was involved in the project as a master of psychology student. All three were trained in qualitative interviewing.** | 11 |
| *Relationship with participants* | | |  |
| 6 | Relationship established | Was a relationship established prior to study commencement?  **The interviewers had no pre-existing relationships with participants.** | 11 |
| 7 | Participant knowledge of the interviewer | What did the participants know about the researcher? e.g. personal goals, reasons for doing the research  **The information sheet for the study outlined that the purpose of the research was to gain a better understanding of the barriers and facilitators to client’s completion of outcome measures and the delivery of feedback on their responses provided by clinicians in AOD services.** | 9 |
| 8 | Interviewer characteristics | What characteristics were reported about the interviewer/ facilitator? e.g. Bias, assumptions, reasons and interests in the research topic  **As above.** | 9 |
| **Domain 2: study design** | | |  |
| *Theoretical framework* | | |  |
| 9 | Methodological orientation and theory | What methodological orientation was stated to underpin the study? e.g. grounded theory, discourse analysis, ethnography, phenomenology, content analysis  **The present study used iterative categorisation.** | 11 |
| *Participant selection* | | |  |
| 10 | Sampling | How were participants selected? e.g. purposive, convenience, consecutive, snowball  **Convenience sample** | 10 |
| 11 | Method of approach | How were participants approached? e.g. face-to-face, telephone, mail, email  **Email.** | 10 |
| 12 | Sample size | How many participants were in the study?  **N=23** | 10 |
| 13 | Non-participation | How many people refused to participate or dropped out? Reasons?  **Participants self-selected to participate in the interviews and no participants dropped out of the interviews.** | N/A |
| *Setting* | | |  |
| 14 | Setting of data collection | Where was the data collected? e.g. home, clinic, workplace  **Via Zoom** | 10 |
| 15 | Presence of non-participants | Was anyone else present besides the participants and researchers?  **No.** | N/A |
| 16 | Description of sample | What are the important characteristics of the sample? e.g. demographic data, date  **In total, N=23 LLW AOD counsellors (18 female; Mage=42.83, SD=11.45) across 11 LLW AOD services opted to participate in the study and completed an interview.**  **Most participants had a background in counselling (30%) or social work (26%), while remaining participants had backgrounds in various areas including psychology and community services. Participants had worked an average of 5.7 years in the AOD treatment sector.** | 10 |
| *Data collection* | | |  |
| 17 | Interview guide | Were questions, prompts, guides provided by the authors? Was it pilot tested?  **Semi-structured qualitative interview with guide followed by the interviewer. The interview was not pilot tested.** | 10 |
| 18 | Repeat interviews | Were repeat interviews carried out? If yes, how many?  **No.** | n/a |
| 19 | Audio/visual recording | Did the research use audio or visual recording to collect the data?  **Yes.** | 10 |
| 20 | Field notes | Were field notes made during and/or after the interview or focus group?  **No.** | n/a |
| 21 | Duration | What was the duration of the interviews or focus group?  **Interview length ranged from 29 to 74 minutes (Mean=50.52, SD=10.32).** | 10-11 |
| 22 | Data saturation | Was data saturation discussed?  **Yes. Interviews were conducted until data saturation (i.e., absence of novel content) was obtained, determined via discussion between the interviewers and research team.** | 10-11 |
| 23 | Transcripts returned | Were transcripts returned to participants for comment and/or correction?  **No.** | n/a |
| **Domain 3: analysis and findings** | | |  |
| *Data analysis* | | |  |
| 24 | Number of data coders | How many data coders coded the data?  **Two. The primary coder (NP) coded all interviews and the secondary coder (MB) coded a subsample of 10 interviews (43%).** | 11 |
| 25 | Description of the coding tree | Did authors provide a description of the coding tree?  **No.** | n/a |
| 26 | Derivation of themes | Were themes identified in advance or derived from the data?  **Themes were identified using a deductive approach (with the semi-structured interview and CFIR implementation framework both serving as a guide).** | 11 |
| 27 | Software | What software, if applicable, was used to manage the data?  **NVivo (Version 14).** | 11 |
| 28 | Participant checking | Did participants provide feedback on the findings?  **No.** | n/a |
| *Reporting* | | |  |
| 29 | Quotations presented | Were participant quotations presented to illustrate the themes / findings? Was each quotation identified? e.g. participant number  **Yes, quotations are presented to illustrate themes and findings. Quotations are identified by participant number.** | 14-18; table 1 |
| 30 | Data and findings consistent | Was there consistency between the data presented and the findings?  **Yes. All data were examined in the context of existing research and the novel contribution to the research.** |  |
| 31 | Clarity of major themes | Were major themes clearly presented in the findings?  **Yes.** |  |
| 32 | Clarity of minor themes | Is there a description of diverse cases or discussion of minor themes?  **Participants generally had converging opinions. Nonetheless, divergence of ideas among study participants is discussed, where applicable.** |  |

# Table S3. Measures and associated items presented to participants as part of the quantitative data

| **Perceptions of PROMs and feedback.**  Stem: Please indicate the extent to which you agree with the following statements…  Response Scale: 1 (strongly disagree) 2 (disagree) 3 (somewhat disagree) 4 (neither agree nor disagree) 5 (somewhat agree) 6 (agree) 7 (strongly agree) |
| --- |
| 1. Outcome Measures take into consideration the needs and preferences of clients |
| 1. Using Outcome Measures improves client care |
| 1. I like the current processes for Outcome Measures completion |
| 1. I think it’s important to collect Outcome Measures at Follow Up for Active Clients |
| 1. I think it’s important to collect Outcome Measures at Follow Up for Inactive Clients |
| 1. Repeated Outcome Measures assessments alone (without any other treatment) are associated with reductions in substance use and related outcomes |
| 1. Clients would be more likely to complete Outcome Measures if they received immediate feedback on their results |
| 1. Clients would be more likely to complete follow-up Outcome Measures if they could easily compare their Baseline and Follow Up results |
| 1. I find the way Outcome Measures results are presented in the feedback form useful for understanding clients’ survey responses |
| 1. I find the way results are presented in the feedback form useful for providing clients feedback on their Outcome Measures |
| **Self-Efficacy to implement PROMs and Feedback**  Stem: Please indicate how confident you are with the following  Response Scale: 1 (not confident) 2 (limited confidence) 3 (somewhat confident) 4 (fairly confident) 5 (very confident) |
| 1. Explaining the purpose of Outcome Measures to clients |
| 1. Motivating clients to complete Outcome Measures |
| 1. Use Outcome Measure results to directly inform your client’s service plan and treatment goals? |
| 1. Following-up clients who are still in treatment to complete Outcome Measures |
| 1. Following-up clients who have left treatment to complete Outcome Measures |
| 1. Providing feedback to a client using the Outcome Measures Feedback? |
| 1. Engage a client in discussion about the Outcome Measure results using the Feedback Form |
| **Organisational and Staff Support for PROMs**  Stem: Please indicate the extent to which you agree with the following statements  Response Scale: 1 (strongly disagree) 2 (disagree) 3 (somewhat disagree) 4 (neither agree nor disagree) 5 (somewhat agree) 6 (agree) 7 (strongly agree) |
| 1. Organisational leadership actively supports Outcome Measures |
| 1. My direct supervisor actively supports Outcome Measures |
| **Available Resources for PROMs** |
| Stem: Please indicate the extent to which you agree the following are available to make outcome measures and brief interventions work in your team  Response Scale: 1 (strongly disagree) 2 (disagree) 3 (somewhat disagree) 4 (neither agree nor disagree) 5 (somewhat agree) 6 (agree) 7 (strongly agree) |
| 1. Equipment and materials (e.g., colour printing) |
| 1. Adequate staff |
| 1. Budget or financial resources |
| 1. Adequate staff time to collect follow-up outcome measures from clients who have not completed them |
| 1. Training |
| 1. Support/Supervision |

*Note.* PROM, patient-reported outcome measures.

Table S4. Staff characteristics and descriptive statistics from quantitative study including all items (*N*=108)

| Variable | M (SD) |
| --- | --- |
| **Knowledge and beliefs regarding PROMs and feedback** | - 1. **(0.93)** |
| 1. Outcome Measures take into consideration the needs and preferences of clients | 5.45 (1.23) |
| 2. Using Outcome Measures improves client care | 5.83 (1.13) |
| 3. I like the current processes for Outcome Measures completion | 4.80 (1.75) |
| 4. I think it’s important to collect Outcome Measures at Follow Up for Active Clients | 5.80 (1.17) |
| 5. I think it’s important to collect Outcome Measures at Follow Up for Inactive Clients | 4.62 (1.64) |
| 6. Repeated Outcome Measures assessments alone (without any other treatment) are associated with reductions in substance use and related outcomes | 3.79 (1.30) |
| 7. Clients would be more likely to complete Outcome Measures if they received immediate feedback on their results | 5.33 (1.16) |
| 8. Clients would be more likely to complete follow-up Outcome Measures if they could easily compare their Baseline and Follow Up results | 5.60 (1.13) |
| 9. I find the way Outcome Measures results are presented in the feedback form useful for understanding clients’ survey responses | 4.66 (1.57) |
| 10. I find the way results are presented in the feedback form useful for providing clients feedback on their Outcome Measures | 4.64 (1.56) |
| **Self-efficacy to implement PROMs and feedback** | **3.93 (0.67)** |
| 1. Explaining the purpose of Outcome Measures to clients | 4.26 (0.80) |
| 2. Motivating clients to complete Outcome Measures | 4.08 (0.86) |
| 3. Use Outcome Measure results to directly inform your client’s service plan and treatment goals? | 4.06 (0.84) |
| 4. Following-up clients who are still in treatment to complete Outcome Measures | 3.99 (0.84) |
| 5. Following-up clients who have left treatment to complete Outcome Measures | 3.06 (1.08) |
| 6. Providing feedback to a client using the Outcome Measures Feedback? | 4.13 (0.82) |
| 7. Engage a client in discussion about the Outcome Measure results using the Feedback Form | 3.94 (0.95) |
| **Leadership support for PROMs** | **6.37 (0.84)** |
| 1. Organisational leadership actively supports Outcome Measures | 6.30 (0.94) |
| 2. My direct supervisor actively supports Outcome Measures | 6.44 (0.93) |
| **Available resources for PROMs** | **5.26 (1.08)** |
| 1. Equipment and materials (e.g., colour printing) | 5.36 (1.50) |
| 2. Adequate staff | 5.28 (1.55) |
| 3. Budget or financial resources | 5.05 (1.50) |
| 4. Adequate staff time to collect follow-up outcome measures from clients who have not completed them | 4.66 (1.66) |
| 5. Training | 5.65 (1.20) |
| 6. Support/supervision | 5.82 (1.09) |

*Notes.* AOD, alcohol and other drugs; PROM, Patient-reported outcome measures. ^a^Some categories were merged due to small cell size to preserve participant anonymity. Range for knowledge and beliefs regarding PROMs and feedback, Leadership Support for PROMs, and available resources for PROMs is 1 (strongly disagree) to 7 (strongly agree). Range for self-efficacy to implement PROMs and feedback is 1 (not confident) to 5 (very confident).

Table S5. Correlations between quantitative study variables (*N*=108)

| Variable | 1. | 2. | 3. | 4. | 5. | 6. | 7. | 8. | 9. |
| --- | --- | --- | --- | --- | --- | --- | --- | --- | --- |
| 1. Age | 1.000 | -0.134 | -0.283 | 0.160 | 0.362 | -0.037 | 0.073 | 0.233 | 0.022 |
| 1. Female |  | 1.000 | -0.029 | 0.022 | -0.032 | 0.020 | -0.025 | -0.012 | 0.128 |
| 1. Highest degree |  |  | 1.000 | -0.035 | -0.144 | -0.041 | -0.056 | -0.047 | -0.004 |
| 1. Length of time working at Lives Lived Well |  |  |  | 1.000 | 0.578 | -0.052 | 0.079 | 0.028 | -0.017 |
| 1. Length of time working in the alcohol and other drug sector |  |  |  |  | 1.000 | -0.083 | 0.188 | 0.046 | 0.073 |
| 1. Knowledge and beliefs regarding PROMs and feedback |  |  |  |  |  | 1.000 | 0.517 | 0.466 | 0.465 |
| 1. Self-efficacy to implement PROMs and feedback |  |  |  |  |  |  | 1.000 | 0.395 | 0.384 |
| 1. Leadership support for PROMs |  |  |  |  |  |  |  | 1.000 | 0.398 |
| 1. Available resources for PROMs |  |  |  |  |  |  |  |  | 1.000 |

*Note.* PROM, Patient-reported outcome measures.
